# Research advances on the risk of prostate cancer from phthalates exposure: from epidemiological evidence to multidimensional prevention and control

**DOI:** 10.3389/fcell.2025.1740894

**Published:** 2026-01-12

**Authors:** Binbin Wang, Hongliang Cao, Shuxin Li, Zhijun Tang, Gengchen Huang, Zhanhao Li, Yutao Ma, Wei Wei, Mo Chen

**Affiliations:** Department of Urology II, The First Hospital of Jilin University, Changchun, China

**Keywords:** biomarkers, endocrine disruption, epidemiological association, mechanism of action, phthalate esters (paes), prevention and control strategies, prostate cancer (PCa)

## Abstract

Prostate cancer (PCa) poses a significant threat to men’s health worldwide, with persistently high incidence and mortality rates. Phthalates (PAEs), typical environmental endocrine disruptors (EDCs), are ubiquitous in the environment and readily accumulate in the human body due to their widespread use in plastics and consumer products. Their potential role in PCa development has drawn considerable attention. This review systematically summarizes the epidemiological associations between PAEs and PCa, their potential mechanisms of action, long-term risks, and corresponding prevention and control strategies. Epidemiological studies confirm that high-molecular-weight PAEs (e.g., di(2-ethylhexyl) phthalate [DEHP], dibutyl phthalate [DBP]) are significantly associated with increased PCa risk, with abdominally obese men identified as a susceptible population. Urinary PAE metabolites (e.g., mono(2-ethylhexyl) phthalate [MEHP], mono-n-butyl phthalate [MnBP]) serve as non-invasive biomarkers for assessing PAE exposure in prostate tissue. Mechanistically, PAEs may regulate PCa progression through multiple pathways, including disrupting the androgen/estrogen signaling balance, inducing epigenetic abnormalities (DNA hypomethylation, microRNA dysregulation), activating pro-proliferative/invasive signaling pathways (MAPK/AP-1, Wnt/β-catenin pathways), and inducing oxidative stress and facilitating epithelial-mesenchymal transition (EMT). Concurrently, PAEs may pose long-term carcinogenic risks through developmental programming and synergistic interactions with obesity to exacerbate PCa risk. Furthermore, this review proposes a multi-tiered prevention and control system comprising industrial source control, targeted protection of susceptible populations, occupational safeguards, and clinical integration. Future research should focus on core scientific questions, such as identifying key PAE subtypes that may be carcinogenic to the prostate, elucidating transgenerational epigenetic mechanisms underlying PAE-induced PCa susceptibility, and verifying the reversibility of PAE-obesity interactions in PCa development, to provide more substantial evidence for mitigating PAE-associated PCa risk.

## 1 Introduction

The prostate, as an accessory male reproductive organ located beneath the bladder, is divided into the central, transitional, and peripheral zones. Malignant proliferation of its epithelial cells may develop into prostate cancer (PCa) (Wilson and Zishiri, 2024). Over 95% of PCa cases are adenocarcinomas, with the vast majority originating from acini rather than ducts, and nearly 80% of prostate adenocarcinomas arise from epithelial cells in the peripheral zone (Wasim et al., 2022). PCa is the most prevalent malignant tumor in the male urinary system (Siegel et al., 2025). In 2020, it ranked second globally in incidence among all cancers (age-standardized incidence rate: 30.7 per 100,000 population) and sixth in mortality (age-standardized mortality rate: 7.7 per 100,000 population), accounting for 7.3% of all global cancer cases and posing a substantial threat to men’s health (Sung et al., 2021). In recent years, its incidence has shown a marked reversal trend, shifting from an annual decline of 6.4% between 2007 and 2014 to a yearly increase of 3.0% from 2014 to 2021 (Kratzer et al., 2025). Studies indicate the average age at PCa diagnosis is 67 years, with over 60% of cases occurring in individuals aged 65 and above (Grozescu and Popa, 2017; Silk et al., 2021). Regarding disease distribution, approximately 75% of cases are localized PCa, 14% involve regional lymph node metastasis, and 10% present with distant metastasis. Bone metastasis is the most common metastatic site, accounting for about 82% of cases (Raychaudhuri et al., 2025). Prognosis varies significantly across disease stages. Patients with early-stage localized PCa diagnosed at an early stage achieve a 10-year survival rate as high as 99%. In contrast, those with advanced (distant metastatic) disease have a 5-year overall survival rate of only 37%, indicating a poor prognosis (Siegel et al., 2025; Rebello et al., 2021). In clinical practice, prostate-specific antigen (PSA)—a glycoprotein secreted by prostate epithelial cells—serves as the most commonly used serum biomarker for PCa screening and diagnosis (Balk et al., 2003; Gao et al., 2025). Patients with mildly elevated PSA levels (4–10 ng/mL) warrant high suspicion for PCa, typically confirmed through transrectal or transperineal prostate biopsy (Raychaudhuri et al., 2025). Despite diverse treatment options and high overall cure rates for PCa, clinical practice faces numerous practical challenges. PSA’s limited diagnostic specificity—elevated levels can result from benign conditions, leading to diagnostic bias—and the inevitable progression of all PCa patients from hormone-sensitive to castration-resistant prostate cancer (CRPC) contribute to diminishing treatment efficacy (Wasim et al., 2022). Even when initial treatment achieves expected outcomes, many patients experience disease recurrence within 10 years (Kustrimovic et al., 2023). Concurrently, PCa is a complex multifactorial disease whose development involves genetic, environmental, and physiological factors (Kustrimovic et al., 2023). However, the specific mechanisms underlying these factors remain not fully elucidated, and preventive intervention systems for primary PCa are still underdeveloped (Wasim et al., 2022). These practical issues require further exploration and breakthroughs.

**FIGURE 1:**
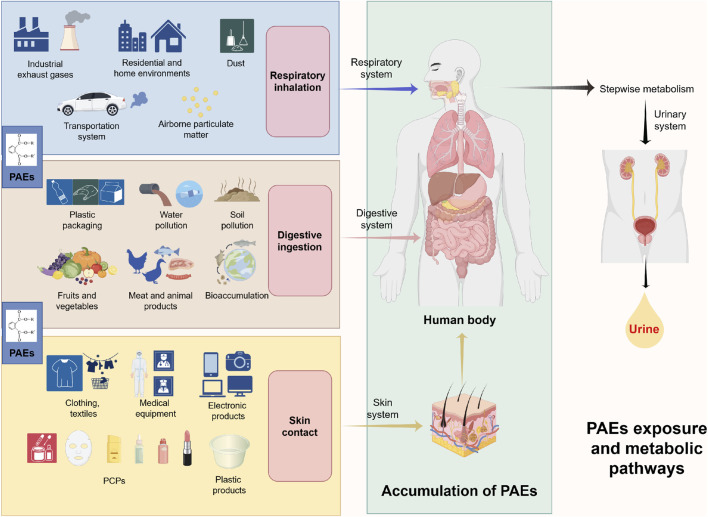
This diagram illustrates the environmental sources of PAEs, human exposure pathways, and their metabolic processes within the body. PAEs are widely used in plastic products, food packaging, medical devices, and products like PCPs, and readily migrate from these products into various environmental media. Human exposure primarily occurs through dermal contact, respiratory inhalation, and ingestion. Additionally, HMW-PAEs resist degradation, enabling bioaccumulation and magnification within aquatic food chains, leading to higher dietary intake of contaminants. After entering the body via various systems, PAEs undergo stepwise metabolism and are primarily excreted in urine. Abbreviations: PAEs, phthalates; HMW-PAEs, high molecular weight PAEs; PCPs, personal care products.

Phthalates (PAEs) are a class of chemicals widely used in industrial production. Their molecular structure consists of diester derivatives of phthalic acid (1,2-benzenedicarboxylic acid), featuring one benzene ring and two ester side chains (Wang Y. et al., 2019; Katsikantami et al., 2016). Based on the length of the leading carbon chain, PAEs can be broadly categorized into low molecular weight PAEs (LMW-PAEs; containing 3-6 carbon atoms) and high molecular weight PAEs (HMW-PAEs; containing 7–13 carbon atoms) (North et al., 2014; Guo T. et al., 2023). Based on structure and application, they can be further categorized into various types, with common examples including di(2-ethylhexyl) phthalate (DEHP), dibutyl phthalate (DBP), butyl benzyl phthalate (BBzP), and diisobutyl phthalate (DiBP) (Wittassek et al., 2011) PAEs are primarily used as plasticizers, solvents, or additives in plastic goods, food packaging, medical devices, and personal care products (PCPs) (Kamrin, 2009). Due to their non-covalent bonding with polyvinyl chloride (PVC), they readily migrate from plastic products into the environment (Rowdhwal and Chen, 2018). Consequently, they are detected in various environmental media, including dust, soil, air, and water bodies. Due to their long half-lives, PAEs have become globally prevalent ecological pollutants (Guo T. et al., 2023; Li et al., 2024). Environmental PAEs can enter the human body through multiple routes, including skin contact, respiratory inhalation, and digestive ingestion (Martínez-Ibarra et al., 2021; Hasan et al., 2025). Furthermore, HMW-PAEs are poorly water-soluble, readily accumulate in aquatic organisms, and undergo biomagnification in the food chain, thereby progressively increasing human exposure (Chatterjee and Karlovsky, 2010; Mondal et al., 2022). Upon entering the body, PAEs are first hydrolyzed by esterases and lipases into monoester metabolites with more vigorous endocrine-disrupting activity (e.g., mono(2-ethylhexyl) phthalate, MEHP) (Pillo et al., 2024; Zhang Y. J. et al., 2021; Hanioka et al., 2017). These primary metabolites, such as glucuronidation, can undergo phase II metabolism to form water-soluble derivatives excreted in urine (Figure 1) (Silva et al., 2003). Consequently, measuring urinary PAEs metabolite concentrations has become the most common method for studying human PAEs exposure (Calafat and McKee, 2006). As typical environmental endocrine disruptors (EDCs), PAEs can participate in various pathological processes such as allergic diseases, reproductive system disorders, and endocrine dysfunction by interfering with endocrine homeostasis and inducing oxidative stress (Katsikantami et al., 2016; Benjamin et al., 2017). Among these, their association with PCa in the urogenital system has garnered considerable attention. Existing research indicates that urinary PAEs metabolites serve as effective, non-invasive biomarkers for assessing actual exposure levels in prostate tissue (Zhang et al., 2025). Population epidemiological studies have indicated a significant association between PAEs exposure and increased PCa risk (Guo T. et al., 2023; Chuang et al., 2020; Yin et al., 2025); animal studies have also demonstrated that PAEs can increase PCa susceptibility through transgenerational effects (Aquino et al., 2023), although the molecular mechanisms underlying this association remain incompletely elucidated. Based on this, this review systematically reviews the epidemiological association and mechanisms of action between PAEs and PCa in the existing literature, aiming to provide a theoretical basis for clarifying the role of environmental exposure in PCa pathogenesis, formulating targeted prevention strategies, and developing novel intervention approaches.

## 2 Exposure evidence: epidemiological association between PAEs and PCa

Epidemiological studies across different populations have established consistent associations between PAEs exposure and PCa risk, supported by evidence from exposure characteristics, dose-response relationships, biomarker validation, and population heterogeneity. These findings provide foundational exposure evidence for the PAEs-PCa association (Table 1).

**TABLE 1:** Observational studies of PAEs and PCa.

Study author and year	Research type	PAEs exposure characteristics	Research subjects/population	Key research findings	References
Chuang et al. (2020)	Population-based nested case-control study (prospective cohort follow-up)	Exposure to DEHP, BBzP, and DiBP quantified via urinary metabolites	Taiwanese Community Population Case Group: 80 PCa cases Control Group: 156 cases	1. Abdominally obese men (waist circumference ≥90 cm) showed a significant positive association between DEHP/BBzP/DiBP exposure and PCa risk; 2. OR for PCa with upper tertile of DEHP metabolites = 7.76 (95% CI: 1.95–30.9); 3. No association in non-abdominally obese men	(Chuang et al., 2020) Environ Res 181: 108902
Guo et al. (2023a)	Population-based cross-sectional study (NHANES data)	Exposure to 13 metabolites (including ∑DEHP, MBzP) quantified via urinary metabolites	U.S. NHANES 2003–2010 Data Total Sample: 1,676 males ≥20 years Case Group: 100 PCa cases Control Group: 1,576 cases	1. ∑DEHP is significantly positively correlated with PCa risk; 2. ∑DEHP is associated with elevated serum PSA in non-PCa individuals; 3. HMW-PAEs metabolites (MBzP, DEHP metabolites) show strong associations with PCa	(Guo et al., 2023a) Reprod Toxicol 116: 108337
Wang et al. (2024a)	Case-control study	Exposure to 9 metabolites (including DEHP, DnBP, DiBP) quantified via urinary metabolites	PCa Group: 187 biopsy-confirmed cases Control Group: 151 cases (PSA <4 ng/mL, no PCa history)	1. DEHP exposure (≥45.42 μg/g creatinine) is significantly associated with PCa (OR = 48.26, 95% CI = 10.74–216.82, p < 0.001); 2. DnBP (MnBP) and DiBP (MiBP) exposure sources differ between cases and controls; 3. Most metabolites have 100% urinary detection rate	(Wang et al., 2024a) J Hazard Mater 474: 134736
Chang et al. (2018)	Case-control study	Exposure to 11 metabolites (including MiNP, MiDP) quantified via urinary metabolites; trace element co-exposure assessed	Central Taiwan Population PCa Group: 20 cases BPH Group: 17 cases Healthy Control Group: 23 cases	1. MiNP is positively associated with PCa risk (OR = 88.02, 95% CI: 3.995–1939, p = 0.005); 2. MiDP is positively associated with BPH risk (OR = 427.32, 95% CI: 2.084–>10000, p = 0.026); 3. MiNP >1 μg/L yields OR = 88.02 for PCa	(Chang et al., 2018) Environ Int 121(Pt 2): 1179–1184
Zhang et al. (2025)	Population cohort - urine-tissue correlation study (PCa patients)	9 PAEs monoesters (MEP, MiBP, MBP, MBzP, etc.) detected in prostate tissue; corresponding urinary metabolites quantified	76 PCa patients (surgical cancer tissue + preoperative urine samples)	1. 4 PAEs monoesters (MEP, MiBP, MBP, MBzP) have 98.68% tissue detection rate; 2. Tissue concentrations (1.12 × 10^-3^–1.86 × 10^2^ ng/g) positively correlate with urinary metabolites; 3. Urinary metabolites are non-invasive biomarkers for prostate tissue exposure	(Zhang et al., 2025) J Sep Sci 48(4): e70154

Abbreviations: PAEs, phthalates; PCa, prostate cancer; DEHP, di(2-ethylhexyl) phthalate; BBzP, butyl benzyl phthalate; DiBP, diisobutyl phthalate; ∑DEHP, sum of DEHP, metabolites; MBzP, monobenzyl phthalate; PSA, prostate-specific antigen; MiNP, monoisononyl phthalate; MiDP, monoisodecyl phthalate; DnBP, dibutyl phthalate; BPH, benign prostatic hyperplasia; MEP, monoethyl phthalate; MiBP, mono-isobutyl phthalate; MnBP, mono-n-butyl phthalate.

### 2.1 Study selection criteria

To ensure rigor and relevance, we applied explicit inclusion and exclusion criteria for study selection: Inclusion criteria focused on human populations with pathologically confirmed PCa outcomes, reliable PAEs exposure assessment via biomonitoring (urinary metabolites or prostate tissue concentrations), quantitative exposure-outcome associations with statistical significance, rigorous study designs (nested case-control, case-control, cross-sectional, or tissue-urine correlation) with sufficient sample size and adjusted confounders, and publication in peer-reviewed journals with accessible full text. Exclusion criteria included animal/*in vitro* studies, lack of quantitative associations, unreliable exposure assessment (e.g., only self-reported contact), and duplicate publications. The selected studies were prioritized for their complementary strengths, covering diverse populations, validating key PAEs subtypes/metabolites, and addressing methodological gaps, providing comprehensive support for the PAEs-PCa association.

### 2.2 Population heterogeneity and susceptible groups

A population-based nested case-control study (80 PCa cases, 156 controls; 20-year follow-up) in Taiwan identified abdominal obesity (waist circumference ≥90 cm) as a key susceptibility factor, among abdominally obese men, exposure to DEHP, BBzP, and DiBP was significantly positively associated with PCa risk, OR for upper tertile of DEHP metabolites = 7.76, 95% CI: 1.95–30.9, while no such association was observed in non-obese men (Chuang et al., 2020). This nested case-control design minimizes Recall bias (exposure assessed pre-diagnosis). However, it has limited generalizability to non-Asian populations, a gap that is partially addressed by larger cohorts in the U.S. and China. The findings highlight that obesity may potentiate PAEs’ effects, emphasizing targeted protection for high-risk subgroups.

### 2.3 Dose-response relationships

A cross-sectional analysis of the National Health and Nutrition Examination Survey (NHANES) 2003–2010 data (1,676 men ≥20 years, 100 PCa cases) confirmed a dose-dependent association: the sum of DEHP metabolites (∑DEHP) correlated positively with PCa risk and elevated serum PSA in non-PCa individuals (both p < 0.05), with all PCa-associated PAEs metabolites showing a “higher concentration, higher risk” trend (Guo T. et al., 2023). A large-scale case-control study (187 PCa cases, 151 controls) further validated this, with the highest ∑DEHP exposure group showing a 48-fold higher PCa risk (adjusted OR = 33.02, p < 0.001) (Wang X. et al., 2024). The NHANES cross-sectional design offers population representativeness but cannot establish temporal causality, while the case-control study strengthens outcome specificity (biopsy-confirmed cases, PSA-negative controls) despite residual confounding risks; consistent dose-response trends across designs reinforce the robustness of the DEHP-PCa association.

### 2.4 Metabolite specificity and biomarker validation

A case-control study (20 PCa cases, 17 benign prostatic hyperplasia [BPH] cases, 23 healthy controls) identified monoisononyl phthalate (MiNP, a DiNP metabolite) as a specific risk indicator. Urinary MiNP levels were significantly higher in PCa patients than healthy controls (p = 0.014), with concentrations >1 μg/L associated with an OR of 88.02 (95% CI: 3.995–1939, p = 0.005) (Chang et al., 2018). Critical validation came from a tissue-urine correlation study in 76 PCa patients, four PAEs monoesters (monoethyl phthalate [MEP], mono-isobutyl phthalate [MiBP], mono-n-butyl phthalate [MnBP], mono-benzyl phthalate [MBzP]) had a 98.68% detection rate in prostate tissue, with tissue concentrations significantly positively correlated with urinary metabolites (Zhang et al., 2025). While the small-scale case-control study is limited by sample size, its findings are indirectly supported by the biomarker validation study, addressing a key limitation of earlier epidemiological research (uncertainty about target tissue exposure) and confirming urinary metabolites as reliable non-invasive proxies for prostate tissue PAEs exposure.

### 2.5 Integrated discussion: epidemiological evidence and link to molecular mechanisms

Collectively, the selected studies confirm three core findings: high-molecular-weight PAEs (DEHP, DBP, BBzP, DiBP) exhibit structure-dependent associations with increased PCa risk; urinary ∑DEHP, MiNP, and MnBP concentrations show dose-dependent correlations with PCa risk, with abdominal obesity potentiating this effect; and urinary MEHP, MnBP, MiBP, and MBzP are validated as non-invasive proxies for prostate tissue exposure. These epidemiological insights provide valuable context for interpreting the molecular mechanisms outlined in Section 3, DEHP’s consistent association with PCa aligns with its documented endocrine-disrupting activity, specifically interference with AR/ER signaling, and its potential to activate oncogenic pathways (MAPK/AP-1, Wnt/β-catenin) in prostate epithelial cells; dose-response trends support the notion that PAEs may act as environmental stressors contributing to carcinogenesis through cumulative cellular perturbations; and the observed PAEs-obesity synergy suggests potential crosstalk between metabolic dysregulation and endocrine disruption, an interplay that may amplify PCa risk. Critically, the validation of urinary biomarkers ensures these epidemiological associations reflect biologically relevant exposure levels in the prostate, addressing a key uncertainty in environmental carcinogenesis research, and thereby supports the plausibility of PAEs’ role in PCa development, while offering an informative framework for connecting population-level observations to the molecular mechanisms detailed in the subsequent section.

## 3 Mechanism evidence: molecular and cellular pathways potentially involved in PCa

EDCs refer to a class of naturally occurring or synthetic exogenous compounds that can disrupt the normal function of endogenous hormones, leading to endocrine system disorders (Lee et al., 2017). Human exposure to EDCs increases the risk of metabolic diseases, developmental and reproductive abnormalities, and endocrine-related cancers (Feijó et al., 2025). Moreover, EDCs can exert biological effects at extremely low concentrations. They may exhibit a “cocktail effect” under mixed exposure conditions, where synergistic enhancement occurs between compounds, complicating their carcinogenic risk assessment (Kumar et al., 2020).

**FIGURE 2:**
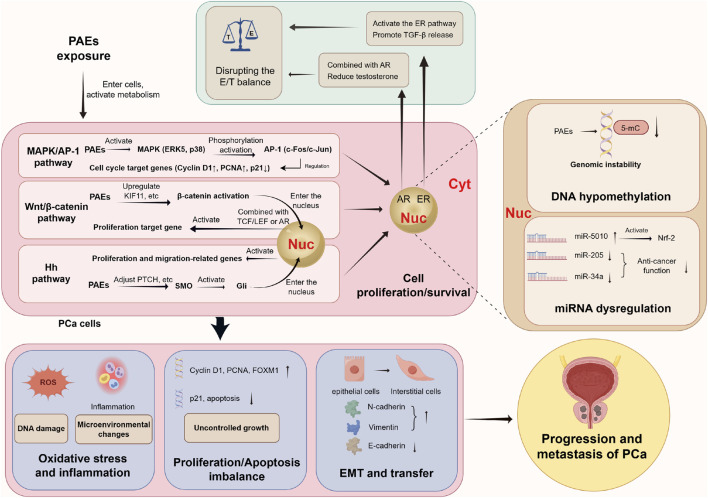
PAEs drive the initiation and progression of PCa by directly binding hormone receptors, activating multiple oncogenic signaling pathways, and inducing epigenetic alterations, ultimately leading to malignant phenotypes such as oxidative stress, proliferation-apoptosis imbalance, and EMT. Abbreviations: PCa, prostate cancer; PAEs, phthalates; Nuc: nucleus; Cyt, cytoplasm; E, estrogen; T, androgen; ER, estrogen receptor; AR, androgen receptor; TGF-β, transforming growth factor-β; MMP-2, matrix metalloproteinase-2; c-myc, transcription factor; cyclin D1, cyclin D1; MAPK/AP-1 pathway, mitogen-activated protein kinase/activated protein C-1 pathway; ERK5, extracellular signal-regulated kinase 5; PCNA, proliferating cell nuclear antigen; TCF/LEF, transcription factor/lymphoid enhancer factor; Hn pathway, Hedgehog pathway; SMO, smoothened protein; Gli, glioma-associated oncogene; DNA, deoxyribonucleic acid; FOXM1, forkhead box M1; 5-mC, 5-methylcytosine; EMT, Epithelial-mesenchymal transition.

Building on the aforementioned epidemiological evidence linking PAEs to PCa, as prototypical EDCs, PAEs may disrupt prostate homeostasis through multiple interconnected molecular and cellular pathways. These include interference with endocrine signaling, regulation of epigenetic modifications, activation of oncogenic signaling cascades, induction of oxidative stress, and perturbation of cell cycle balance. Additionally, PAEs may enhance long-term carcinogenic potential by interacting with developmental programming processes and metabolic pathways. Collectively, these multifaceted effects are thought to contribute to the initiation and progression of PCa (Figure 2).

### 3.1 Primary potential trigger: disruption of androgen/estrogen (E/T) signaling balance

The prostate is a sex steroid-sensitive gland dependent on balanced androgen (T) and estrogen (E) signaling for homeostasis (De Falco and Laforgia, 2021). Consequently, any substance capable of disrupting T or E signaling pathways (such as EDCs) may disturb prostate homeostasis, induce pathological changes, and ultimately potentially contribute to PCa progression (Lacouture et al., 2022). Research indicates that an elevated E/T ratio plays a significant role in the development of PCa (Wang et al., 2017). PAEs, as EDCs, disrupt this balance via “anti-androgenic” or “estrogen-mimetic” activities, potentially initiating or exacerbating prostate epithelial cell abnormalities.

#### 3.1.1 Androgen pathway dysregulation

Androgen receptor (AR) signaling is pivotal for prostate epithelial cell proliferation, and its dysregulation is a central driver of prostatic carcinogenesis (Grubisha and DeFranco, 2013). PAEs, particularly HMW-PAEs such as DEHP, interfere with this pathway through multiple mechanisms. DEHP binds AR with an affinity comparable to its natural ligand, testosterone, while its primary metabolite, MEHP, shares a 91%–100% similarity with testosterone in key AR amino acid residue interactions (Beg and Sheikh, 2020). This structural mimicry enables MEHP to compete for AR binding and effectively disrupt canonical androgen signaling. Furthermore, DEHP exposure reduces systemic testosterone levels by disrupting the hypothalamic-pituitary-testicular axis and upregulating 5α-reductase two expression (Ha et al., 2016). This combination of direct receptor antagonism and reduced ligand availability creates a sustained perturbation of androgen signaling.

However, the long-term biological effects of this perturbation may involve more than transient signal inhibition. Chronic interference with AR signaling and the resultant hormonal imbalance could foster a pro-inflammatory microenvironment and genomic instability in pre-malignant lesions (Mimeault and Batra, 2013). This sustained anti-androgenic pressure, analogous to clinical androgen deprivation therapy, may paradoxically select for adaptive cellular clones (e.g., through AR amplification or alternative pathway activation), potentially contributing to progression toward CRPC (Cheng et al., 2022; Zhou et al., 2021). Thus, PAEs may exert immediate anti-androgenic effects while also promoting long-term adaptations associated with more aggressive disease phenotypes (Zhang T. et al., 2021).

Observational evidence from animal studies provides support for this proposed framework. DEHP exposure has been shown to dose-dependently elevate the estradiol (E2)/T ratio, upregulates AR and estrogen receptor α (ERα) expression, and enhance cyclooxygenase-2 (COX-2) signaling, a pathway strongly linked to PCa progression (Zhou et al., 2022; Tian et al., 2019). The upregulation of COX-2 leads to increased prostaglandin synthesis, which in turn promotes cancer cell proliferation, increases inflammatory response, and stimulates angiogenesis within the tumor microenvironment, thereby potentially promoting cancer progression (Zhou et al., 2022; Xia et al., 2018). However, it is important to note that current evidence is primarily derived from controlled experimental models. Future longitudinal studies integrating real-world exposure assessment with molecular epidemiology are needed to elucidate these mechanisms in human populations further and clarify the role of individual genetic and epigenetic factors in determining susceptibility.

#### 3.1.2 Estrogenic effects and stroma-epithelium crosstalk

Prostate tissue primarily expresses two ER subtypes: ERα and ERβ. PCa tissue exhibits characteristic ERα overexpression and ERβ downregulation (Yang and Chen, 2004; Yang et al., 2007), with ERα mediating pro-proliferative effects and ERβ exerting tumor-suppressive functions (Bonkhoff, 2018; Nelson et al., 2014). PAEs function as ERα agonists and ERβ antagonists, exacerbating this subtype imbalance (Guo T. et al., 2023). For example, DBP (with weak estrogenic activity) activates ERα to upregulate pro-proliferative genes (c-myc, cyclin D1) and downregulate cyclin-inhibiting gene p21, potentially accelerating LNCaP cell cycle progression (Lee et al., 2014; Di Lorenzo et al., 2018). ERα activation also promotes PCa cell inflammation, deteriorating the prostate microenvironment (Di Zazzo et al., 2019). In stromal cells, PAEs-induced estrogen-ERα binding upregulates matrix metalloproteinase-2 (MMP-2) and releases transforming growth factor-β (TGF-β), which acts paracrinally on epithelial cells to enhance invasiveness, forming a stroma-epithelium interactive loop potentially associated with cancer promotion and progression (Lee et al., 2014; Yu et al., 2011).

### 3.2 Potential persistent regulation: epigenetic modifications

PAEs induce heritable epigenetic changes (without altering DNA sequences) that may persistently regulate PCa-related gene expression, providing a foundation for long-term tumor development and progression.

#### 3.2.1 DNA hypomethylation

Genomic DNA hypomethylation causes genetic instability and tumor heterogeneity (Pogribny and Beland, 2009; Guo H. et al., 2023; Ehrlich, 2009). Research indicates that the DEHP metabolite MEHP dose-dependently (1–25 μmol/L) reduces whole-genome 5-methylcytosine (5-mC) levels in LNCaP cells, disrupting DNA methylation homeostasis and altering methylation patterns of tumor-related genes to potentially promote cancer cell growth and metastasis (Wu et al., 2017; Liang et al., 2025). Furthermore, paternal DEHP exposure also disrupts sperm DNA methylation, leading to abnormal methylation of embryonic development-related genes—epigenetic changes that are transgenerationally heritable and may increase offspring prostate susceptibility to carcinogens (Nowak et al., 2024; Oluwayiose et al., 2021).

#### 3.2.2 MicroRNA (miRNA) dysregulation

miRNAs are endogenous small non-coding RNAs that play a key regulatory role in cancer proliferation and invasion through post-transcriptional gene silencing (C and ho, 2007). Exposure to PAEs can dysregulate miRNA expression profiles, thereby potentially driving PCa progression. For instance, a clinical controlled study demonstrated that DEHP exposure upregulates miR-5010 and downregulates miR-205 in PCa. The increased miR-5010 activates the Nrf-2 signaling pathway and its downstream targets, enhancing tumor invasiveness (Tsai et al., 2025). Conversely, miR-205 acts as a tumor suppressor by regulating apoptosis, inhibiting proliferation, and modulating epithelial-mesenchymal transition (EMT); DEHP-mediated downregulation of miR-205 thus may facilitate PCa progression (Chauhan et al., 2022). In addition, another controlled study reported that BBzP downregulates the tumor-suppressive miR-34a, upregulating its target gene c-myc to potentially promote cell proliferation via the miR-34a/c-myc axis (Zhu et al., 2019). Furthermore, elevated urinary levels of MEHP have been associated with reduced miR-106a expression, impairing semen quality and potentially contributing to transgenerational carcinogenicity (Cui et al., 2022).

### 3.3 Potential signal amplification: abnormal activation of pro-proliferative/invasive signaling pathways

PAEs can target and activate several core signaling pathways in PCa cells. Through a cascade involving upstream kinase phosphorylation, downstream transcription factor activation, and target gene expression, PAEs may directly contribute to tumor cell proliferation, survival, and invasion (Figure 2).

#### 3.3.1 MAPK/AP-1 pathway

PAEs activate the mitogen-activated protein kinase/activator protein-1 (MAPK/AP-1) pathway, which potentially promotes PCa cell proliferation (Mileo et al., 2023). The MAPK network, including ERK1/2, JNK, p38, and ERK5, serves as a critical upstream regulator of AP-1 transcription factors, influencing processes such as proliferation, differentiation, and stress responses (Whitmarsh, 2007). AP-1 complexes (e.g., c-Fos/c-Jun) act as key downstream effectors in PCa progression. *In vivo* studies using Nkx3.1; Pten mice revealed that Jun and Fos mRNA levels are low in normal prostate and low-grade prostatic intraepithelial neoplasia (PIN), but increase significantly in androgen-dependent high-grade PIN/cancer (2.8-fold and 3.9-fold, respectively) and further rise in advanced androgen-independent lesions (4.8-fold and 8.3-fold; Ouyang et al., 2008). *In vitro* evidence confirms that both c-Jun and c-Fos are upregulated in metastatic PCa, with high c-Jun expression correlating with a poor prognosis (Mileo et al., 2023). Functionally, c-Jun promotes proliferation partly through paracrine stimulation of insulin-like growth factor-1 (IGF-1) synthesis, which subsequently acts on epithelial IGF-1 receptors to drive cell growth (Riedel et al., 2021; Niu and Xia, 2009). At the mechanistic level, PAEs such as DEHP, BBP, and DBP specifically activate ERK5 and p38, upregulate AP-1 (c-Fos/c-Jun), enhance expression of proliferation-related genes (Cyclin D1, PCNA), and suppress the cell-cycle inhibitor p21, collectively potentially driving abnormal PCa cell proliferation (Zhu et al., 2018). Moreover, methoxyacetic acid (MAA), a major active metabolite of PAEs, can enhance ER(α/β) transcriptional activity via MAPK activation, potentially synergistically enhance carcinogenic risk (Parajuli et al., 2015).

#### 3.3.2 Wnt/β-catenin pathway

The Wnt/β-catenin pathway is a central regulator of cell proliferation, apoptosis, and EMT, playing a key role in cancer initiation and progression (Arend et al., 2013). PAEs activate this pathway through multiple mechanisms. DEHP, for example, downregulates the tumor suppressor KLF7 (Kruppel-like factor 7), thereby releasing transcriptional repression on β-catenin and potentially promoting PCa cell proliferation and metastasis (Li et al., 2023). Additionally, DEHP upregulates KIF11 expression, forming a “KIF11-β-catenin” axis that may drive invasion (Song et al., 2024). Consequently, KIF11 levels have been proposed as a potential biomarker for assessing PCa invasiveness and prognosis (Piao et al., 2017). Notably, nuclear β-catenin can interact with T-cell factor/lymphoid enhancer factor (TCF/LEF) transcription factors and directly bind ligand-activated AR, forming a functional “β-catenin-TCF-AR” transcriptional complex. This complex amplifies the expression of androgen-responsive genes such as PSA, thereby potentially enhancing AR-driven tumor growth—a mechanism particularly relevant in CRPC (Corti et al., 2022; Schneider et al., 2018).

#### 3.3.3 Hedgehog pathway

The Hedgehog (Hh) pathway is crucial in embryonic development, and its aberrant activation is closely linked to tumorigenesis and metastasis in multiple cancers, including PCa (Song et al., 2022; Katoh and Katoh, 2008). Normally, Sonic hedgehog (Shh) binding to Patched1 releases inhibition of Smoothened (SMO), leading to activation of the terminal transcription factor Gli (Petrov et al., 2021). In PCa, Gli regulates cell proliferation and is associated with tumor heterogeneity and bone metastasis (Yang et al., 2017; Zhang et al., 2022). PAEs can disrupt this pathway by targeting downstream components. For instance, MEHP upregulates PTCH, a key gene in the Hh pathway, and activates Hh signaling in LNCaP cells (Yong et al., 2016). Interestingly, the classic SMO inhibitor cyclopamine fails to block this activation, suggesting that MEHP acts downstream of SMO and may induce resistance to Hh-targeted therapies (Yong et al., 2016). Additionally, at the molecular level, MEHP exhibits a high affinity for targets such as FOXS1, which binds Hh pathway transcription factor Gli1 to inhibit its ubiquitination and degradation, stabilizing Gli1 and potentially promoting PCa cell growth and metastasis (Wang and Huang, 2023).

### 3.4 Contribution to malignant cellular phenotypes

PAEs may drive PCa progression by inducing oxidative stress, disrupting the balance between proliferation and apoptosis, and promoting key phenotypic transitions. Collectively, these alterations enable PCa cells to acquire aggressive traits, enhancing their proliferation, survival, and metastatic capacity.

#### 3.4.1 Oxidative stress and inflammation

Upon exposure, DEHP is rapidly metabolized into active intermediates that disrupt mitochondrial function, leading to excessive reactive oxygen species (ROS) production and elevated oxidative stress (Tetz et al., 2013; Yang et al., 2021). This redox imbalance is further exacerbated by the inhibition of key antioxidant enzymes, such as glutathione peroxidase 1 (GPx1), which compromises cellular defense mechanisms and leads to structural damage to DNA and lipids in LNCaP cells (Guo T. et al., 2023). Studies indicate that prolonged exposure (24–72 h) to a mixture of PAE metabolites not only increases ROS production and upregulates oxidative stress-related genes, but also enhances the migratory capacity of these cells (Cavalca et al., 2022). Further studies indicate that, in PCa patients, PAEs exposure correlates with elevated oxidative stress markers and may promote tumor invasiveness by activating the Nrf-2 pathway and its downstream effectors (Tsai et al., 2025).

#### 3.4.2 Proliferation-apoptosis imbalance

PAEs disrupt cellular homeostasis by simultaneously promoting proliferation and impairing apoptotic signaling. Zhao et al. demonstrated that DEHP exposure interferes with DNA damage repair pathways, reduces repair efficiency and leads to cell cycle arrest (Zhao et al., 2022). Its metabolite MEHP produces similar effects in both cellular and animal models, impairing DNA double-strand break repair and increasing micronucleus formation, thereby potentially contributing to genomic instability over time (Sun et al., 2022; Silva et al., 2022). Additionally, DEHP upregulates β-catenin while downregulating tumor suppressors such as p53 and KLF7, further potentially stimulating proliferation and metastatic potential (Li et al., 2023). Clinical studies reveal that serum from PAE-exposed PCa/BPH patients shows significantly elevated levels of lipid peroxidation marker MDA and DNA oxidation marker 8-OH-dG, accompanied by increased rates of DNA strand breaks (Chang et al., 2018).

Beyond interfering with DNA repair, studies indicate that the transcription factor FOXM1 represents another key target through which PAEs may enhance proliferative signaling (Liang et al., 2025; Jawwad et al., 2025). MEHP promotes PCa cell growth by upregulating FOXM1, which regulates PSA transcription and supports multiple oncogenic processes, including sustained proliferation, evasion of cell death, and activation of invasion, particularly in androgen-independent (AI) PCa (Liu et al., 2017; Lee et al., 2024).

#### 3.4.3 EMT and metastatic dissemination

EMT is a fundamental process during which epithelial cells lose polarity and intercellular adhesion while acquiring mesenchymal characteristics, thereby enhancing motility and invasiveness (Son and Moon, 2010; Pastushenko and Blanpain, 2019). This transition is molecularly characterized by downregulation of E-cadherin and upregulation of mesenchymal markers such as N-cadherin and vimentin (Xu et al., 2022).

Research indicates that PAEs may promote EMT through multiple signaling axes. Activation of the aryl hydrocarbon receptor (AhR) pathway by PAEs inhibits E-cadherin transcription while inducing expression of N-cadherin, vimentin, and matrix metalloproteinases (MMPs), thereby potentially facilitating cellular detachment and invasion (Akbariani et al., 2025; Shan et al., 2020; Debnath et al., 2022). Independently, PAE-induced upregulation of the oncogene FOXS1 enhances EMT through upregulation of hypoxia-induced lipid droplet-associated protein (HILPDA), which activates the FAK/PI3K/AKT signaling cascade and further may drive metastatic progression in PCa (Ren et al., 2024). Together, these mechanisms illustrate how PAEs modify PCa cells structurally and functionally to support dissemination.

## 4 Long-term risks potentially associated with PAEs exposure

**FIGURE 3:**
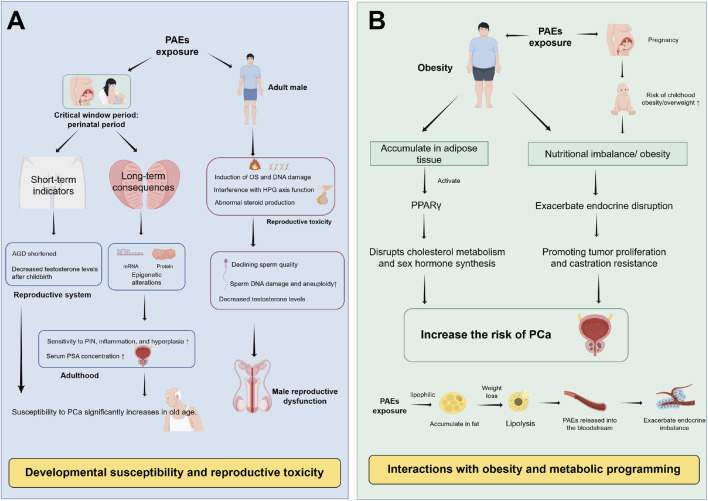
Long-term risk pathways of PAEs potentially associated with PCa. **(A)** Developmental susceptibility: Perinatal PAEs exposure programs prostate development and endocrine function, shortens AGD, and induces transgenerational epigenetic changes, may increase adult PCa susceptibility. **(B)** Obesity synergy: PAEs may promote obesity via PPARγ activation, and obese adipose tissue enhances PAEs’ bioaccumulation and estrogen synthesis, forming a synergistic loop that may enhance PCa risk. Abbreviations: PAEs, phthalates; AGD, anogenital distance; PCa, prostate cancer; PIN, Prostatic intraepithelial neoplasia; PSA, prostate-specific antigen; OS, oxidative stress; DNA, deoxyribonucleic acid; HPG axis, hypothalamic-pituitary-gonadal axis; PPARγ, peroxisome proliferator-activated receptor γ.

Beyond direct molecular-cellular mechanisms, PAEs may pose long-term PCa risks through two distinct but synergistic pathways: developmental programming (transgenerational susceptibility) and metabolic interactions (synergy with obesity). These pathways expand the susceptible population and extend the time window of risk, forming critical components of the PAEs-PCa evidence chain (Figure 3).

### 4.1 Developmental susceptibility and transgenerational toxicity

Early life stages constitute a critical “sensitive window” for organ development, during which exposure to environmental toxins can induce permanent alterations (Basak et al., 2020). Animal studies indicate that perinatal (gestational and lactational) exposure to PAEs disrupts mRNA and protein expression profiles in the ventral prostate of offspring, potentially increasing susceptibility to PCa and other prostatic pathologies, including prostatic intraepithelial neoplasia (PIN), proliferative inflammatory atrophy, and hyperplasia, in aged animals (Aquino et al., 2023; Xia et al., 2018; Peixoto et al., 2016). Supporting this, a study by Xiu Wang et al. demonstrated that DEHP exposure (0.01–1 mg/kg) from gestation day 7 to postnatal day 21 dose-dependently reduced prostate weight in rat offspring. By adulthood (postnatal day 196), these animals exhibited significantly increased PIN incidence, higher Gleason scores, and elevated serum PSA levels, illustrating a clear “developmental-senescence” continuum of potential carcinogenic susceptibility (Wang et al., 2017). Furthermore, multiple studies indicate that prenatal PAEs exposure can lead to shortened anogenital distance (AGD) in male infants and reduced postnatal testosterone levels (Swan et al., 2005; Morová et al., 2020). Epidemiological studies confirm that longer AGD correlates with lower PCa risk (OR = 0.83, 95% CI: 0.70–0.99), suggesting that AGD may serve as a potential epidemiological marker linking fetal PAEs exposure to PCa risk (Castaño-Vinyals et al., 2012).

Beyond transgenerational risk, paternal PAEs exposure disrupts sperm DNA methylation and impairs reproductive function, which may further influence offspring susceptibility. Through mechanisms involving oxidative stress, DNA damage, disruption of the hypothalamic-pituitary-gonadal (HPG) axis, and hormone-like effects, PAEs compromise steroidogenesis and metabolic balance (Høyer et al., 2018). Research by Jurewitz et al. demonstrated that elevated urinary levels of PAE metabolites correlate with reduced sperm motility, lower testosterone, and increased sperm DNA damage and aneuploidy (Jurewicz et al., 2013). DEHP exposure has also been shown to impair human sperm quality and accelerate sperm biological aging directly (Wang Y. X. et al., 2019; Oluwayiose et al., 2022). However, the effects of chronic low-dose exposure remain controversial and warrant further population-based investigation.

### 4.2 Synergistic interaction with obesity

Nutritional imbalance and obesity represent significant health challenges in modern society. Existing research indicates that obesity is an established risk factor for aggressive PCa (Gharieb et al., 2024). PAEs’ exposure interacts synergistically with obesity to potentially amplify carcinogenic risk. Epidemiological studies reveal a positive correlation between prenatal PAEs exposure and childhood obesity/overweight status (Shree et al., 2022). Research by Akritidis et al. indicates that DEHP and its metabolites correlate positively with overall obesity (Akritidis et al., 2025). Notably, PAEs’ lipophilic nature facilitates accumulation in adipose tissue, where they can activate peroxisome proliferator-activated receptor gamma (PPARγ), thereby disrupting cholesterol metabolism and sex hormone synthesis (Chuang et al., 2020; Del Río Barrera et al., 2025; Elix et al., 2018).

This interaction creates a vicious cycle. In obesity, increased adipose tissue aromatase activity elevates estradiol levels. This effect synergises with the inherent estrogenic properties of PAEs, further disrupting the E/T balance and potentially driving PCa proliferation and castration-resistant progression (Chuang et al., 2020). Furthermore, PAEs stored in adipose tissue can be released into the bloodstream during weight loss, exacerbating endocrine disruption (Dirtu et al., 2013). Thus, the “PAE exposure → obesity → enhanced PAE bioaccumulation → amplified PCa risk” cycle highlights a critical pathway through which metabolic status and environmental chemical exposure jointly may influence cancer susceptibility.

## 5 Multilevel prevention and control strategies for PCa potentially associated with PAEs exposure and prospects for clinical intervention

**FIGURE 4:**
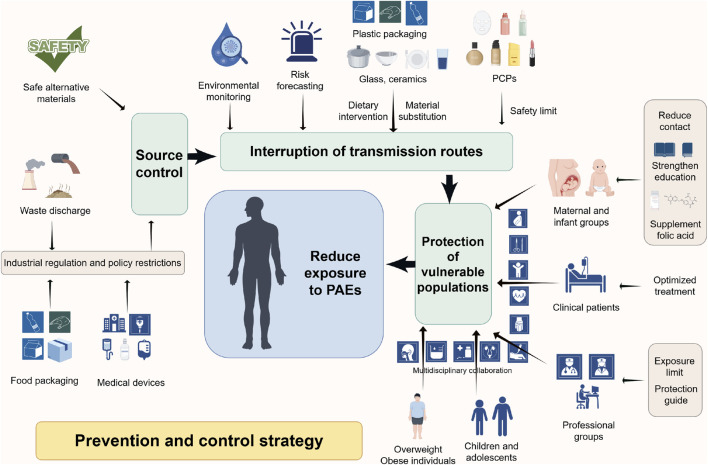
This diagram systematically summarizes the multi-level prevention and control strategies proposed to reduce the health risks associated with PAEs exposure. The core strategy is based on PAEs exposure prevention and control measures following the principle of “source control - pathway interruption - protection of susceptible populations.” Industrial regulation and policy restrictions reduce PAEs production and use at the source; advanced technologies for environmental monitoring and risk prediction, safety threshold setting, dietary interventions, and safe material substitutions block exposure pathways such as oral and dermal exposure; enhanced maternal and child healthcare, occupational protection, and patient management prioritize high-risk populations, ultimately establishing a multidisciplinary, comprehensive prevention and control system. Abbreviation: PAEs, phthalates; PCPs, personal care products.

As typical EDCs, PAEs may drive the development and progression of PCa through multifaceted mechanisms, including endocrine disruption, epigenetic dysregulation, and aberrant activation of oncogenic signaling pathways. Their long-term risks, such as transgenerational susceptibility from perinatal exposure and synergistic interactions with conditions like obesity, further underscore a significant public health challenge. Given their pervasive presence in industrial products, food packaging, and daily consumer goods, population-wide exposure is complex and difficult to avoid entirely. Notably, the “Developmental Origins of Health and Disease (DOHaD)” hypothesis highlights that environmental exposures during critical windows of susceptibility (preconception, prenatal, perinatal, and childhood) can permanently reprogram organ development and metabolic homeostasis via epigenetic modifications, thereby increasing the risk of adult-onset diseases, including PCa (Ho et al., 2017). Evidence indicates that PAE exposure during sensitive life stages can induce persistent molecular alterations and increase long-term PCa susceptibility (Aquino et al., 2023; Burns et al., 2022). Whereas in adults, cumulative exposure may synergise with factors such as obesity to amplify risk further. Given this gradient of susceptibility across the lifespan, reducing PAE exposure through targeted interventions during critical periods can block early pathogenic cascades, whereas controlling exposure in adulthood helps prevent additional risk accumulation. Therefore, establishing a comprehensive, multi-tiered prevention and control system, encompassing source control, targeted protection for key populations, nationwide exposure reduction, and clinical integration, is critical for reducing the potential disease burden of PCa potentially associated with PAEs (Figure 4).

### 5.1 Source control: reducing environmental PAEs emissions and promoting alternatives

Preventing the environmental release of PAEs forms the foundational layer of the prevention system, directly determining overall population exposure levels. Legislative action is essential to restrict the use of HMW-PAEs (e.g., DEHP, DBP), which are closely associated with PCa risk, in high-exposure products such as food packaging and medical devices (Zhou et al., 2025). Concurrently, industrial emissions of PAEs in waste gasses, wastewater, and residues must be strictly regulated to reduce pollution at its source. Environmental and biological monitoring can leverage advanced technologies, such as ultra-high-performance liquid chromatography-tandem mass spectrometry (UPLC-MS/MS), to establish a PAEs exposure surveillance network. This enables precise quantification of PAE metabolite levels in urine and tissues, providing crucial data for tracing regional pollution and assessing population risk (Zhang et al., 2025). The integration of computational approaches, such as molecular docking simulations, further supports the development of early warning systems in high-risk areas (Zhang T. et al., 2021; Liu et al., 2025). In the long term, developing and validating safe alternative materials is paramount. Efforts should be accelerated to promote substitutes like triethyl acetyldimonium citrate (ATEC), which exhibit no estrogenic or anti-androgenic activity (Xu et al., 2019; Park et al., 2019). Evidence confirms that policy interventions restricting PAEs in consumer products can significantly reduce urinary metabolite concentrations in the population, thereby indirectly lowering potential PCa risk associated with PAEs exposure (Sieck et al., 2024).

### 5.2 Targeted protection of key susceptible populations

Building on source control, implementing targeted protection for populations with heightened vulnerability is essential to maximize risk reduction. Maternal-fetal populations, as core targets for PAEs’ transgenerational effects, require special attention since perinatal exposure alters offspring prostate mRNA and protein expression profiles, potentially increasing adult PCa susceptibility (Aquino et al., 2023). While human epidemiological data link prenatal PAEs exposure to shortened AGD (Lu et al., 2024). Pregnant women should be advised to minimize contact with disposable plastic products, and PAEs exposure risks should be incorporated into standard prenatal health education. Patients undergoing PCa treatment must be aware that PAEs can interfere with therapeutic efficacy; for instance, DBP may promote tumor progression by competitively binding to the AR or activating ERα, potentially undermining androgen deprivation therapy (ADT) (Zhang T. et al., 2021). Therefore, integrating exposomics monitoring into clinical management is advisable. For instance, urine samples could be collected during procedures like robot-assisted prostatectomy to monitor PAEs metabolite levels dynamically, informing personalized therapy adjustments (Tsai et al., 2025).

Furthermore, occupational groups such as plasticizer manufacturers, plastic processors, healthcare workers, and firefighters face elevated PCa risk due to prolonged exposure at high concentrations (Eckert et al., 2023; Park et al., 2024; Kolena et al., 2020; Janjani et al., 2024; Shi et al., 2024; Shi et al., 2023; Nguyen et al., 2022). Tailored protective standards for these groups are necessary, including defined exposure limits, provision of chemical-resistant personal protective equipment, and the establishment of routine biomonitoring programs. Linking occupational exposure data to PCa incidence in a dedicated database would support evidence-based updates to safety guidelines.

### 5.3 Nationwide interventions for daily exposure reduction

Expanding interventions to the general population can further reduce daily exposure risks through integrated public education, policy, and lifestyle modifications. Public health education should target high-exposure scenarios and priority groups. For children and adolescents who frequently use plastic products, studies show significant correlations between urinary PAE metabolite levels and health risks, necessitating advocacy for reducing reliance on single-use plastics (Wang Y. et al., 2024). Specific consumer products require management, as the use of some sunscreens has been linked to elevated urinary levels of mono-n-hexyl phthalate (MnHxP), likely due to the presence of di-n-hexylphthalate (DnHxP)/MnHxP (Frederiksen et al., 2025). Regulatory bodies can establish safety thresholds, as exemplified by the EU Scientific Committee on Consumer Safety (SCCS), which recommends a maximum limit of 260 ppm for DnHxP as a byproduct, or below 1 ppm if present as an unavoidable impurity (Scherer et al., 2025). Dietary intervention is another critical measure, as approximately 90% of human PAEs exposure stems from contaminated water or food (Wang et al., 2023). Studies indicate that high PAEs exposure indices are typically associated with consuming takeout food, using plastic containers and packaging, and employing children’s cosmetics and bath products (Jung et al., 2025). Therefore, dietary habits should be adjusted to reduce intake of processed foods, thereby decreasing exposure to HMW-PAEs, or substitute PCPs to minimize exposure to LMW-PAEs (Ma et al., 2019; Wu et al., 2025; Yang et al., 2023). Additionally, glass or ceramic containers should be prioritized over plastic containers for food preparation and storage (Hall and Greco, 2019). Research also suggests folate supplementation may reduce urinary PAEs metabolite concentrations (Huang et al., 2025). Women planning or during pregnancy are advised to consume folate through diet or supplements to mitigate PAEs accumulation (Chen et al., 2025).

### 5.4 Clinical integration: towards a prevention-diagnosis-treatment closed loop

Fostering multidisciplinary collaboration between environmental medicine and oncology is essential to integrate PAEs exposure assessment into the routine care pathway for PCa, thereby achieving a closed-loop management system from prevention to treatment. For instance, incorporating urinary PAEs metabolite testing (e.g., MEHP, MnBP) into preoperative evaluations and postoperative follow-ups for PCa patients, combined with PSA levels and Gleason scores, can dynamically adjust treatment plans. Particularly for patients undergoing ADT, drug dosages should be optimized based on exposure levels. Given the DOHaD-derived risk of transgenerational susceptibility, clinical counseling for PCa patients with a history of early-life PAEs exposure (e.g., maternal use of plastic products during pregnancy) should include recommendations for offspring to minimize PAEs exposure and to undergo regular PCa screening. Future efforts should prioritize longitudinal studies to validate the efficacy of these integrated interventions. The ultimate goal is to progressively establish an individualized risk-warning system, informed by pharmacogenomics and continuous biomonitoring. This approach aims to achieve comprehensive risk management spanning from pollution control at the source to endpoint patient protection, ultimately improving outcomes for PAEs-exposed PCa patients.

## 6 Future research directions

Building on current evidence and identified knowledge gaps, future research should prioritize several core scientific questions to strengthen the causal link between PAE exposure and PCa and to inform targeted prevention and clinical intervention strategies.

### 6.1 Identification of key carcinogenic PAEs and their mechanisms in mixed exposures

While epidemiological studies have highlighted associations between specific high-molecular-weight PAEs (e.g., DEHP, DBP) and PCa risk, real-world exposure involves complex mixtures of multiple PAEs and other EDCs (Liang et al., 2025). Future work should focus on identifying which PAE subtypes play pivotal roles in prostate carcinogenesis through large-scale prospective cohorts and multi-center validations. This includes quantifying the independent and combined contributions of specific PAEs metabolites to PCa initiation and progression, and elucidating potential synergistic, additive, or antagonistic interactions in “cocktail” exposure scenarios. For instance, studies have shown that mixtures of PAE metabolites can induce oxidative stress and enhance migratory capacity in prostate cancer cells (Cavalca et al., 2022), while co-exposure to PAEs and trace elements may exacerbate oxidative damage in PCa patients (Chang et al., 2018). Additionally, interactions between PAEs and bisphenols (another class of EDCs) have been implicated in PCa occurrence, highlighting the need for comprehensive mixture risk assessment (Wang X. et al., 2024).

Developing integrated exposure assessment tools that combine environmental monitoring, biomonitoring (e.g., urine, blood, tissue PAEs metabolites), and dietary surveys will be essential for accurately characterizing individual cumulative exposure. For example, simultaneous detection of PAEs monoesters in prostate tissue and urine has provided reliable biomarkers for exposure assessment (Zhang et al., 2025), which can be expanded to mixed exposure scenarios to refine risk prediction models.

### 6.2 Elucidating the transgenerational epigenetic pathways in PAEs-Induced PCa susceptibility

Although animal studies indicate transgenerational carcinogenic risks following perinatal PAEs exposure, the specific epigenetic mechanisms underlying this heritable susceptibility in humans remain poorly defined (Wang et al., 2017). Future research should decipher the patterns of transgenerational epigenetic inheritance, focusing on alterations in DNA methylation, histone modifications, and non-coding RNA profiles (e.g., miRNAs, lncRNAs, circRNAs), induced by maternal and paternal PAEs exposure (Ho et al., 2017).

Preclinical studies have provided critical clues: maternal exposure to PAE mixtures can alter miRNA expression and transcriptome profiles in offspring prostate tissue, linking developmental exposure to PCa risk (Aquino et al., 2023; Aquino et al., 2024). Specifically, *in utero* and lactational DEHP exposure increases prostate carcinogenesis susceptibility in offspring by inducing PSCA hypomethylation (Xia et al., 2018), whereas MEHP can induce DNA hypomethylation in LNCaP cells, disrupting genomic stability (Wu et al., 2017). Longitudinal cohort studies tracking these epigenetic changes across generations, coupled with functional validation in experimental models, are needed to identify stable epigenetic markers associated with increased PCa risk. The ultimate goal is to integrate these epigenetic signatures with conventional risk factors (e.g., age, family history, obesity) to construct early-warning models that identify individuals at high lifetime risk of PCa.

### 6.3 Investigating the reversibility of PAEs-Obesity interactions and targeted interventions

The synergistic interplay between PAEs exposure and obesity appears to amplify PCa risk, but whether this interaction is reversible remains an open question. Existing evidence confirms that combined exposure to PAEs and a high-fat diet can induce histopathological alterations in the prostate (de Jesus et al., 2015), while PAE exposure correlates with abdominal obesity and may disrupt lipid metabolism, thereby exacerbating carcinogenicity (Akritidis et al., 2025).

Future studies should explicitly test the reversibility of metabolic-chemical interactions through well-designed intervention trials in obese populations with documented high PAEs exposure. Key inquiries include whether weight loss, dietary modifications, or increased physical activity can reduce PAE body burden, restore endocrine homeostasis, and mitigate associated cancer risks. Concurrently, the potential of nutritional supplements to reverse PAEs-induced epigenetic dysregulation should be evaluated: population-based studies have shown that serum and red blood cell folate concentrations are associated with reduced urinary PAEs metabolite levels (Huang et al., 2025), while folic acid and vitamin D may modulate the adverse effects of prenatal PAEs exposure (Chen et al., 2025). These efforts will inform the development of personalized intervention protocols for PCa patients and high-risk individuals, combining exposure reduction, metabolic management, and adjuvant therapies to improve outcomes.

### 6.4 Translational integration: from mechanistic insights to clinical and policy applications

To bridge the gap between mechanistic understanding and real-world impact, future research must foster closer integration of basic science with clinical and public health practice.

At the therapeutic level, translating key mechanistic findings into targeted strategies is critical. For example, MEHP-induced PCa progression involves activation of the Hh pathway and Wnt/β-catenin signaling, providing potential targets for small-molecule inhibitors (Li et al., 2023; Yong et al., 2016). Network toxicology and molecular docking approaches have identified key interaction targets of PAEs metabolites in PCa cells, laying the groundwork for drug development (Liang et al., 2025). Additionally, incorporating urinary PAEs metabolites and relevant epigenetic biomarkers into PCa screening, diagnosis, and prognostic assessment could enhance early detection and risk stratification. For instance, urinary MiNP levels >1 μg/L are associated with a significantly increased PCa risk (Chang et al., 2018), while specific miRNA signatures (e.g., miR-34a, miR-205) altered by PAEs exposure may serve as complementary biomarkers (Tsai et al., 2025; Zhu et al., 2019).

At the policy level, evidence from population-based intervention studies should directly inform regulations: policy restrictions on PAEs use in consumer products have been shown to reduce urinary metabolite concentrations (Sieck et al., 2024), supporting the promotion of safer alternatives (e.g., citrate ester plasticizers) to achieve primary prevention at the population level (Xu et al., 2019).

## 7 Conclusion

This review synthesizes the core evidence linking PAEs to PCa. Epidemiologically, high-molecular-weight PAEs (e.g., DEHP, DBP) are significantly associated with PCa risk, with abdominally obese individuals as susceptible groups, and urinary metabolites (e.g., MEHP, MnBP) serving as reliable non-invasive biomarkers. Mechanistically, PAEs may drive PCa via disrupting androgen/estrogen balance, inducing epigenetic abnormalities, activating oncogenic pathways (MAPK/AP-1, Wnt/β-catenin, Hedgehog), and promoting malignant phenotypes (oxidative stress, EMT). Long-term risks include transgenerational susceptibility from perinatal exposure and synergistic risk amplification with obesity. The proposed multi-tiered prevention system (source control, susceptible population protection, clinical integration) provides a practical framework for risk mitigation. Future research should prioritize identifying key PAEs subtypes, clarifying transgenerational epigenetic mechanisms, verifying the reversibility of PAE-obesity interactions, and advancing translational applications. This work offers valuable insights for reducing the PAE-associated PCa burden and safeguarding men’s health.
